# Phrenology and Napoleon

**Published:** 1842-11

**Authors:** 


### PHRENOLOGY AND NAPOLEON.

Napoleon's bust, as it is to be seen in phrenological cabinets, must have struck every one as very unlike the head of a great warrior. In fact, it is a very *mediocre* thing, and we have always felt, that, if it represented faithfully the size and form of the emperor's head, it was exceedingly out of place in a phrenological cabinet, since it set fairly at defiance every principle of organology. We have heard various attempts at explaining the discrepancy. It has been said, that, after Napoleon's exile to St. Helena, his brain shrunk from inaction, and that, consequently, the bust taken after his death does not give the proportions of his head as it was in the prime of his life. Another explanation is, that Antomarchi was an *anti-phrenologist,* and published a false bust of his illustrious patient. The true account of the matter is probably given in the following anecdote, which we lately met with in a biographical sketch of Dr. Graves, of Dublin, and for which we are sure he will receive the thanks of all phrenologists.

"When Napoleon died, there was no plaster of Paris in St. Helena, and no one who knew how to take a plaster bust of the emperor, which he had never allowed any one to do during his life (why, it is difficult to say). The emperor's suite expressed great regret at this circumstance, in the presence of Doctor Burton of the 66th regiment, a relative of Dr. Graves (who had all the particulars from him). Doctor Burton had assisted in the post-mortem examination of Napoleon—his name is to the attested certificate of that investigation—and when he heard these expressions of regret, he offered to make all possible efforts to form a bust, on the condition, that if he succeeded he should have the original, and the emperor's attendants a copy. He had in so hot a climate, to hasten his operations, and was out all night in a boat seeking sulphate of lime (gypsum), which he had previously observed in veins on the rocks near the shore. He collected a sufficient quantity, prepared it, and after much labor succeeded in taking an excellent cast of Napoleon's bust. Next evening he went to town, having labored all day, to take some refreshment in his quarters, leaving the mould at Longwood. Some of the officers, hearing from him what he had done, said he would never see the mould again. To this he answered, that he did not believe any effort would be made to rob him of his rights, which he had taken care to verify by a written contract; but when he returned to Longwood, he found the mask or face part of the mould had been stolen. The rest was left—its use either being not understood, or being too heavy to get away privately. No entreaties of Dr. Burton were availing to recover the mask. Madame Bertrand said she would never permit an "Englishman to *publish* the emperor." This mask was published by Antomarchi, Napoleon's physician, and not only the mask but the whole bust. Except the mask, every other part was fictitious, as Doctor Burton secured the rest, and yet throughout Europe, the imaginary bumps, in this supposed cast of Napoleon's cranium, have proved quite satisfactory to phrenologists as affording undoubted proofs of the accuracy and truth of their science. Doctor Burton died suddenly on parade, at Canterbury, while doing duty with a regiment of hussars to which he had been appointed surgeon; and the mould, there is too much reason to believe, was destroyed in his quarters; his servants being ignorant of its value." Y.

